# Report of Committee on Mortality and Hygiene, Made to Chicago Medical Society April 7th, 1851

**Published:** 1851-05

**Authors:** W. B. Herrick

**Affiliations:** Chairman


### ARTICLE V.

*Report of the Committee on Mortality and Hygiene, made to the Chicago Medical Society April 7th,* 1851, *and ordered to be published in the N. W. Medical and Surgical Journal.*

The members of this Society are aware that there are no official records kept of the mortality of this city, and that therefore, it is extremely difficult to obtain the requisite number of facts upon which to make a proper report upon this subject.

The statistical information herewith presented, gives the number of annual interments for eight years from 1843 to 1850. Also the comparative mortality of adults and children for each month, and the number of deaths from cholera from 1846 to 1850, inclusive.

From 1843 to 1848, the annual mortality of Chicago ranged from 1 in 34.61 to 1 in 64.78, making the average proportion of deaths during that period less than that of most other cities of the United States. In 1849 the mortality was 1 in 18.84, and in 1850 1 in 21.45, making the deaths during these two cholera seasons in the proportions, nearly, of two to one more than at any previous period.

By an examination of the table showing the comparative mortality at different seasons, it will be seen that during the winter and spring months the fatality is greatest amongst adults; whilst in the summer and fall there are proportionally more deaths of children, excepting during the cholera seasons, in which the mortality of adults is in excess during the whole year. W. B. HERRICK, *Chairman.*

Table No. I. Population, deaths and rate of mortality in Chicago from 1846 to 1850, inclusive.

| Year. | Populat'n. | Mortal'y fr'm cholera | Mort'y fr'm var's dis'es | Of these adults | Of these children | Annual mortl'y | Mortal'y 1 in ev'y | Mortality perc'tage |
|---|---|---|---|---|---|---|---|---|
| 1846 | 14,169 | | 327 | 154 | 173 | 327 | 43.33 | 2.30 |
| 1847 | 16,859 | | 487 | 270 | 217 | 487 | 34.61 | 2.88 |
| 1848 | 19,724 | | 514 | 228 | 286 | 514 | 38.37 | 2.60 |
| 1849 | 23,047 | 681 | 542 | 787 | 436 | 1223 | 18.84 | 5.30 |
| 1850 | 28,620 | 463 | 871 | 743 | 591 | 1334 | 21.45 | 4.66 |
| | 114507 | | | | | 4175 | 27.67 | 3.65 |

Table No. II. Comparative mortality of Children and Adults in Chicago, at different seasons, from 1846 to 1850, inclusive.

| | 1846. | | 1847. | | 1848. | | 1849. | | 1850. | | Pro'tion. | | Tot. |
|---|---|---|---|---|---|---|---|---|---|---|---|---|---|
| Months. | Adults | Ch'ren | A | C | A | C | A | C | A | C | A | C | |
| January, | 10 | 13 | 12 | 13 | 12 | 12 | 20 | 24 | 40 | 15 | 94 | 77 | 171 |
| February, | 6 | 9 | 11 | 8 | 14 | 12 | 21 | 17 | 21 | 30 | 73 | 76 | 149 |
| March, | 8 | 9 | 16 | 12 | 17 | 13 | 20 | 8 | 24 | 20 | 85 | 62 | 147 |
| April, | 5 | 5 | 12 | 8 | 13 | 16 | 29 | 13 | 20 | 22 | 79 | 64 | 143 |
| May, | 11 | 10 | 14 | 10 | 23 | 19 | 60 | 24 | 20 | 22 | 128 | 85 | 213 |
| June, | 4 | 8 | 17 | 8 | 10 | 21 | 85 | 36 | 32 | 27 | 148 | 100 | 248 |
| July, | 11 | 18 | 22 | 21 | 16 | 27 | 223 | 84 | 169 | 86 | 441 | 236 | 677 |
| August, | 27 | 40 | 33 | 56 | 17 | 40 | 158 | 115 | 262 | 212 | 497 | 463 | 960 |
| Sept., | 16 | 23 | 37 | 42 | 25 | 35 | 86 | 53 | 76 | 75 | 240 | 228 | 468 |
| October, | 32 | 28 | 32 | 23 | 35 | 25 | 46 | 31 | 38 | 32 | 183 | 139 | 322 |
| Nov., | 11 | 15 | 39 | 10 | 29 | 50 | 22 | 19 | 24 | 18 | 125 | 112 | 237 |
| Dec., | 13 | 8 | 15 | 6 | 17 | 16 | 17 | 12 | 17 | 32 | 79 | 74 | 153 |

Table. No. III. Population, deaths and rate of mortality in Chicago for eight years from 1843 to 1850, inclusive.

| Year. | Population. | Annual mortality | Mortality, one in every | Percentage of mortality. |
|---|---|---|---|---|
| 1843 | 7,580 | 117 | 64.78 | 1.54 |
| 1844 | 10,170 | 288 | 38.78 | 2.83 |
| 1845 | 12,088 | 290 | 41.68 | 2.39 |
| 1846 | 14,169 | 327 | 43.33 | 2.30 |
| 1748 | 16,859 | 487 | 34.61 | 2.88 |
| 1848 | 19,724 | 514 | 38.37 | 2.60 |
| 1849 | 23,047 | 1,223 | 18.84 | 5.30 |
| 1850 | 28,620 | 1,334 | 21.45 | 4.66 |

